# Catalase protects *Aedes aegypti* from oxidative stress and increases midgut infection prevalence of Dengue but not Zika

**DOI:** 10.1371/journal.pntd.0005525

**Published:** 2017-04-05

**Authors:** José Henrique M. Oliveira, Octávio A. C. Talyuli, Renata L. S. Goncalves, Gabriela Oliveira Paiva-Silva, Marcos Henrique F. Sorgine, Patricia Hessab Alvarenga, Pedro L. Oliveira

**Affiliations:** 1 Laboratório de Bioquímica de Artrópodes Hematófagos, Instituto de Bioquímica Médica Leopoldo de Meis, Programa de Biologia Molecular e Biotecnologia, Universidade Federal do Rio de Janeiro, Rio de Janeiro, Brasil; 2 Instituto Nacional de Ciência e Tecnologia em Entomologia Molecular (INCT-EM), Brasil; 3 Laboratório de Bioquímica de Resposta ao Estresse, Instituto de Bioquímica Médica Leopoldo de Meis, Programa de Biologia Molecular e Biotecnologia, Universidade Federal do Rio de Janeiro, Rio de Janeiro, Brasil; North Carolina State University, UNITED STATES

## Abstract

**Background:**

Digestion of blood in the midgut of *Aedes aegypti* results in the release of pro-oxidant molecules that can be toxic to the mosquito. We hypothesized that after a blood meal, the antioxidant capacity of the midgut is increased to protect cells against oxidative stress. Concomitantly, pathogens present in the blood ingested by mosquitoes, such as the arboviruses Dengue and Zika, also have to overcome the same oxidative challenge, and the antioxidant program induced by the insect is likely to influence infection status of the mosquito and its vectorial competence.

**Methodology/Principal findings:**

We found that blood-induced catalase mRNA and activity in the midgut peaked 24 h after feeding and returned to basal levels after the completion of digestion. RNAi-mediated silencing of catalase (AAEL013407-RB) reduced enzyme activity in the midgut epithelia, increased H_2_O_2_ leakage and decreased fecundity and lifespan when mosquitoes were fed H_2_O_2_. When infected with Dengue 4 and Zika virus, catalase-silenced mosquitoes showed no alteration in infection intensity (number of plaque forming units/midgut) 7 days after the infectious meal. However, catalase knockdown reduced Dengue 4, but not Zika, infection prevalence (percent of infected midguts).

**Conclusion/Significance:**

Here, we showed that blood ingestion triggers an antioxidant response in the midgut through the induction of catalase. This protection facilitates the establishment of Dengue virus in the midgut. Importantly, this mechanism appears to be specific for Dengue because catalase silencing did not change Zika virus prevalence. In summary, our data suggest that redox balance in the midgut modulates mosquito vectorial competence to arboviral infections.

## Author summary

Mosquitoes ingest large amounts of blood, a rich and abundant source of energy to sustain egg production. Blood digestion offers challenges to the insect, like managing high concentrations of heme and iron, pro-oxidant and potentially toxic molecules derived from hemoglobin. Mosquitoes and other blood-feeding arthropods have evolved adaptations to overcome this problem, such as the activation of catalase, an antioxidant enzyme that protect tissues against toxic free radicals. Mosquitoes act as important vectors of human diseases because during a blood meal they might also ingest microorganism circulating in our blood, such as dengue and zika virus. The adaptive antioxidant program that protects mosquito tissues against the oxidative challenge imposed by a blood meal might also influences the ability of virus to establish infection and disseminate from the midgut to the salivary glands. We show here that catalase differentially influences the number of infected midguts after mosquitoes were challenged with blood contaminated with virus, being beneficial to Dengue-4 but neutral do Zika, suggesting that redox metabolism may have distinct roles on mosquito vector competence towards different arbovirus.

## Introduction

Arthropod-borne viral (arboviral) diseases, such as Dengue, Chikungunya, and especially Zika, have recently occupied a central spot in the global discussions concerning infectious diseases due to the rapid spread of cases worldwide and the associated increase in syndromes such as microcephaly and Guillain–Barré, prompting the World Health Organization to declare Zika a public health emergency [1]. Over two million people live in areas where Zika has been reported, highlighting the risk of major epidemics, especially in Central and South Americas, as well as Southeast Asia [2]. Concerning Dengue, more accurate epidemiological data are available, and the number of annual infections could be as high as half a billion [3]. Currently, there is no vaccine to prevent new infections and no effective treatment options for sick individuals. Strategies targeting *Aedes aegypti*, the mosquito vector, such as utilizing the anti-viral effects of Wolbachia [4,5] and the sterile insect technique [6,7], are attractive possibilities under implementation. To stop mosquito spread of arboviral diseases, we need to further elucidate the molecular interactions between the virus and the vector. This knowledge will help to explain, for example, the observed differences in susceptibility to viral infections of mosquito strains/populations[8,9].

Reactive oxygen species (ROS) have emerged as central molecules in a wide array of pathological as well as physiological processes, including signaling, immunity, cell proliferation and differentiation [10]. The biological actions of ROS are based on their ability to donate or receive electrons from biomolecules, triggering a diverse set of events associated with the normal function of cells. However, under oxidative stress, elevated levels of ROS may disrupt redox signaling pathways, leading to a non-homeostatic state commonly associated with disease [11]. Therefore, the correct balance between ROS-generating systems (such as mitochondria, endoplasmic reticulum or NADPH oxidases) and ROS-detoxifying reactions (including antioxidant enzymes such as catalase, which detoxifies H_2_O_2_ into water and oxygen) is critical for maintaining homeostasis in virtually all studied organisms.

ROS metabolism influences critical parameters of insect physiology, including fecundity [12,13], immune response [14,15] and vector competence in the interaction between *Anopheles* and *Plasmodium* [16–22]. In hematophagous arthropods, such as the *Aedes aegypti* mosquito, blood digestion in the midgut releases heme, a pro-oxidant molecule. Cells subjected to high concentrations of heme, such as gut epithelial cells after a blood meal, must maintain redox balance to avoid oxidative stress. Blood-sucking organisms have evolved a series of adaptations against the deleterious effects of excess heme [23–29]. One such mechanism is the activation of antioxidant enzymes, such as catalase, which reduces H_2_O_2_ and prevents its contact with heme/iron, a reaction known to generate highly toxic ROS [30,31]. An interesting possibility concerning hematophagous insect vectors is that the antioxidant protection induced upon blood feeding could also defend human pathogens being carried by the mosquitoes, such as arboviruses, from blood-induced oxidative challenge.

Our results demonstrated that a blood meal up-regulated catalase mRNA and activity in the midgut epithelium and that silencing of catalase through RNAi reduced mosquito fecundity and resistance to hydrogen peroxide feeding. Catalase silencing had no effect on the intensity of infection with Dengue or Zika viruses. Interestingly, it reduced the prevalence of Dengue infection, but had no effect on the prevalence of Zika-infected females. This indicates that the redox environment of the midgut can alter mosquito susceptibility to infection with some flaviviruses.

## Materials and methods

### 2.1 –Ethics statement

All animal care and experimental protocols were conducted in accordance with the guidelines of the Committee for Evaluation of Animal Use for Research of the Universidade Federal do Rio de Janeiro (CEUA-UFRJ). The protocols were approved under the registry CEUA-UFRJ 155/13. Dedicated technicians at the animal facility at the Institute of Medical Biochemistry (UFRJ) carried out all aspects related to rabbit husbandry under strict guidelines to ensure careful and consistent handling of the animals.

### 2.2 –Mosquitoes

Two– 10-day-old *Aedes aegypti* females (red-eye strain) were used in all the assays and were maintained in 12-h light-dark periods at 28°C and 80% relative humidity. Females were fed *ad libitum* with cotton pads soaked in a 5% sucrose solution or allowed to feed on rabbit blood.

### 2.3 –Catalase activity

Mosquitoes were cold-anesthetized and dissected in 50% ethanol. The midgut epithelia was separated from the blood bolus and collected in PBS (10 mM sodium phosphate buffer and 150 mM NaCl, pH 7.4) supplemented with a protease inhibitor cocktail (50 μg/mL SBTI, 1mM benzamidine, 1mM PMSF). Samples were mechanically homogenized with a pestle and stored at -80°C until use. Catalase activity was measured following H_2_O_2_ absorbance (240 nm for 1 min) according to the protocol described by Aebi [32] in the presence of mosquito homogenates. The protein concentration was determined according to Lowry [33]. For *in vivo* inhibition experiments, mosquitoes were fed blood supplemented with different doses of 3-amino-1,2,4-triazole (AT), a catalase inhibitor. For *in vitro* inhibition, AT was incubated for 30 minutes at 4°C with tissue homogenates before measurements of enzymatic activity.

### 2.4 –RNA extraction and quantitative PCR (qPCR)

Total RNA (pools of 5–10 midgut epithelia) was extracted with TRIzol reagent (Invitrogen) according to the protocol suggested by the manufacturer and treated with DNAse I. cDNA was synthesized using a High-Capacity cDNA Reverse Transcription Kit (Applied Biosystems) according to standard protocols. The sequence CAT1A –AAEL013407 –RB (Transcript ID from VectorBase) was used to design the catalase primers. The primer sequences used for qPCR experiments were CAATGAACTGCACCGACAAC (forward) and AGCCTCATCCAGAACACGAC (reverse). The sequence AAEL003396-RA (Transcript ID from VectorBase) was used as a housekeeping gene and corresponded to the ribosomal gene rp49 [34]. The corresponding primers for the housekeeping gene were GCTATGACAAGCTTGCCCCCA (forward) and TCATCAGCACCTCCAGCT (reverse). qPCR was performed using the SYBR Green PCR Master Mix (Applied Biosystems). Relative gene expression was calculated using the method described by Livak and Schmittgen [35].

### 2.5 –RNAi silencing of catalase

A 1158-base-pair fragment of the catalase gene (AAEL013407 –RB) was amplified using cDNA from the midgut epithelia of blood-fed mosquitoes (24 h after feeding) using primers F-TTCAAGGAGTCCCAGAAGGA and R-AACCGGAATCAGAGGGAACT. This amplicon was subjected to a nested PCR with catalase primers that also contained a T7 binding sequence (which is necessary for RNA polymerase binding, see underlined). The primers used were TAATACGACTCACTATAGGGACTCCACTTGCTGTGCGTTT (forward) and TAATACGACTCACTATAGGGTCTCCCTTAGCAATAGCGTTG (reverse). A 773-base-pair fragment was generated, purified and used as the template for an *in vitro* transcription reaction for the synthesis of double-stranded RNA (dsRNA) for catalase (dsCat) with a MEGAscript RNAi kit (Ambion). RNAi experiments were performed via injection of 69 nL of dsCat (3 μg/μL) (or dsLacZ as an unrelated dsRNA) in the thoraxes of 2-day old female mosquitoes. Mosquitoes were used 2–3 days after the injections. A 218-bp LacZ fragment was amplified using the primers GAGTCAGTGAGCGAGGAAGC (forward) and TATCCGCTCACAATTCCACA (reverse) and was cloned into the pCRII-TOPO vector. This plasmid was used for a subsequent PCR in which the T7 RNA polymerase promoter was also inserted, using the primer sequences GTAAAACGACGGCCAGT (M13F) and CTCGAGTAATACGACTCACTATAGGGCAGGAAACAGCTATGAC (M13R). This PCR product was used for the synthesis of the dsLacZ performed with a MEGAscript RNAi kit (Ambion).

### 2.6 –Hydrogen peroxide measurements

H_2_O_2_ was measured with Amplex Red (Invitrogen) following the recommendations of the manufacturer with minor modifications. The midgut epithelia of sugar-fed mosquitoes were dissected in 2.5% BSA in PBS, the gut contents were washed out and the epithelia (pools of 5 organs) were incubated in PBS under dim light at room temperature in the presence of Amplex Red (40 μM) and 4 U horseradish peroxidase (HRP, Sigma). After a 30 min incubation, the epithelia were centrifuged, and the supernatants were collected and evaluated for fluorescence emission at 530/590 nm (Ex/Em) in a Varian Cary Eclipse Fluorescence Spectrofluorometer. The resulting values were subtracted from fluorescence readings generated by nonspecific Amplex Red oxidation by the midgut epithelia (pools of 5 organs) in the absence of HRP.

### 2.7 –Dengue 4 and Zika virus stocks

Dengue-4 was kindly provided by Dr. João Trindade Marques (UFMG–Universidade Federal de Minas Gerais, Brazil). Zika virus was obtained from Dr. Laura Helena Vega Gonzales Gil (Centro de Pesquisas Aggeu Magalhães, Fundação Oswaldo Cruz, Brazil). Viral stocks were propagated in C6/36 cells maintained in Leibovitz-15 (L-15) media (Gibco #41300–039) pH 7.4 supplemented with 5% fetal bovine serum, triptose 2.9 g/L, 10 mL of 7.5% sodium bicarbonate/L; 10 mL of 2% L-glutamine/L, 1% of non-essential amino acids (Gibco #11140050) and 1% penicillin/streptomycin at 28°C. Culture supernatants containing viral particles were harvested, centrifuged, aliquoted and stored at -80°C until use. Plaque assays (see section 2.8) were performed to determine viral titers. The Dengue-4 titer used was 1 x 10^7^/mL. The Zika titer used was 2 x 10^7^/mL.

### 2.8 –Mosquito infection with Dengue-4 and Zika virus

Mosquitoes were starved from sucrose (but not water) for 18–24 h and were offered a meal containing a 1:1 mix of rabbit red blood cells and L-15 media containing different amounts of Dengue-4 or Zika virus. ATP pH 7.4 at a final concentration of 1 mM was included as a phagostimulant. Viral stocks were thawed immediately before use. Mosquitoes were allowed to ingest the infectious blood through a membrane attached to an artificial feeder kept at 37°C for approximately 40 min inside a BSL-2 insectary facility. Mosquitoes were quickly cold-anesthetized, and fully engorged females were separated and housed as indicated in section 2.1 until use.

### 2.9 –Plaque assays

Dengue-4 plaque assays were performed in BHK-21 cells and Zika plaque assays were performed in Vero cells maintained in DMEM (Gibco #12100–046) supplemented with sodium bicarbonate, 1% L-glutamine (200 mM, Gibco #25030081), 10% fetal bovine serum and 1% penicillin/streptomycin and seeded as monolayers (approximately 70% confluency) onto 24-well plates 12–24 h before the experiment. Seven days after the infectious meal, mosquitoes were surface-sterilized with 70% ethanol (20 seconds) and rinsed twice with sterile PBS. Midguts were dissected using clean glass slides and forceps in sterile PBS and transferred to sterile Eppendorf tubes contain 200 μl of DMEM (same as above) and 50–100 mg of sterile glass beads (Scientific Industries SI-BG05–0.5 mm diameter). Midguts were individually stored at -80°C until use. Midgut tissue was disrupted to liberate viral particles by vortexing the tubes for 10 minutes at room temperature. The samples were then centrifuged at 10,000 x *g* at 4°C and serially diluted in DMEM. One hundred μl of each sample was added to Vero (Zika) or BHK-21 (Dengue-4) cell culture monolayers and gently shaken for 15 minutes at room temperature, followed by an additional 45 minutes without shaking at 37°C and in a 5% CO_2_ incubator. Subsequently, 700 μl of DMEM containing 2% FBS and 0.8% methylcellulose (Sigma #M0512. Viscosity 4,000 cP) was added to each well. Plates were incubated at 37°C and 5% CO_2_ for five days. Samples were stained with a 1% crystal violet solution in a 1:1 (v:v) mixture of methanol/acetone for 1 h at room temperature and washed with water to remove excess dye. Then, individual plaque forming units (PFU) were visually counted.

### 2.10 –Statistical analysis

All experiments were carried out independently at least two times, and statistical analyses were performed with GraphPad Prism software. The appropriate tests are described in the figure legends.

## Results

### Blood feeding transiently induced catalase activity in the midgut epithelia

**Fig 1:**
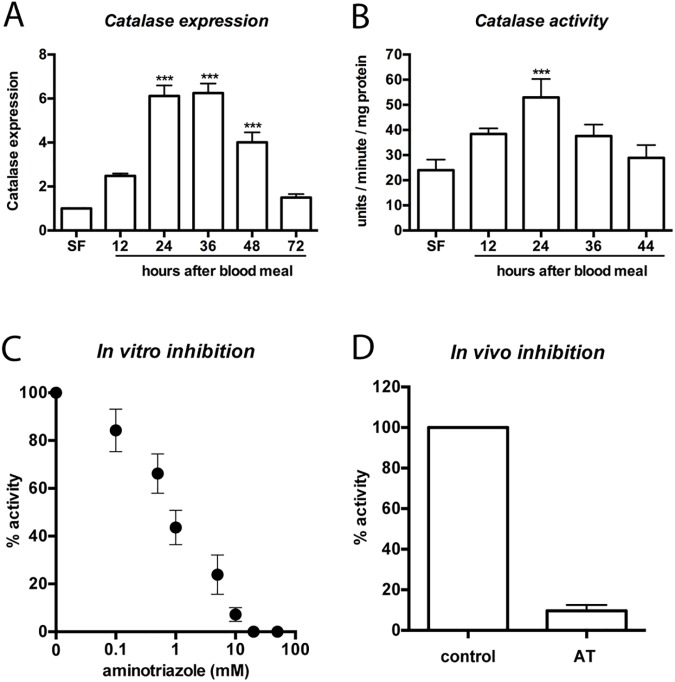
Catalase mRNA and activity increased in the midgut epithelia of blood-fed mosquitoes. *Aedes aegypti* females were fed sugar (SF) or blood (BF) and dissected at the indicated time points after a blood meal. (A) Catalase mRNA expression in the epithelia was evaluated using qPCR analysis of SF and BF mosquitoes. (B) Enzymatic activity was measured as described in the Materials and Methods section. (C) Catalase inhibition by AT *in vitro*. Midgut epithelia was collected from blood-fed mosquitoes 24 h after feeding and was incubated with AT and H_2_O_2_ for 30 min at 4°C; then, catalase activity was assayed. (D) Mosquitoes were fed blood supplemented with 15 mM AT and assayed for catalase activity in the epithelia *** *p <* 0.001. Figure 1A-B–ANOVA followed by Dunnett's multiple comparison test.

To investigate the role of catalase in the midgut of *Aedes aegypti* in response to blood feeding, we compared gene expression in the epithelia of sugar-fed (SF) and blood-fed (BF) females dissected at 12, 24, 36, 48 and 72 h after blood intake. Catalase mRNA levels increased 6-fold at 24 and 36 h after a meal and decreased to SF levels at 72 h (Fig 1A). Enzyme activity was also monitored throughout the digestion process, which spans approximately 48 h. Fig 1B shows that H_2_O_2_-removing capacity also increased in the epithelia after feeding, reaching its maximal induction at 24 h, near the peak of blood digestion [36], and returned to initial levels at 44 h.

We tested the sensitivity of catalase to 3-amino-1,2,4-triazole (AT), a well-known inhibitor [37]. Using an *in vitro* assay exposing midgut samples collected 24 h after a blood meal to different concentrations of AT, we showed that 100% of the epithelial H_2_O_2_ detoxification could be abrogated with AT concentrations below 20 mM and that 50% enzyme inhibition occurred close to 1 mM. Using a similar approach, we fed females blood supplemented with 15 mM AT and showed that catalase activity accounted for more than 90% of H_2_O_2_ removal 24 h after the meal (Fig 1D).

### RNAi silencing of catalase reduced oviposition and resistance to H_2_O_2_

**Fig 2:**
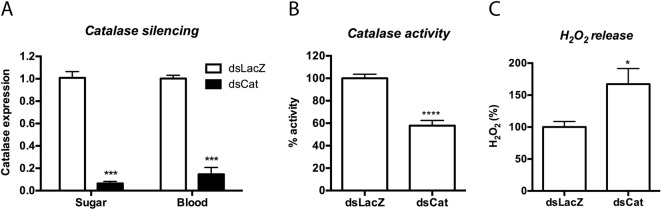
Catalase knockdown in the midgut. (A) Two-day-old females were injected with dsRNA against catalase (dsCat) or an unrelated control gene (dsLac). Two days after the dsRNA injection, a group of mosquitoes were fed blood, while others were fed exclusively with sugar. Twenty-four hours later, RNA was extracted for qPCR analysis. (B) Catalase activity in the epithelia was measured 24 h after a blood meal in dsRNA-treated mosquitoes. (C) Hydrogen peroxide leakage was measured in the midgut epithelia of SF mosquitoes injected with dsCat or dsLacZ. (A) *** *p <* 0.001 (t-test). (B) **** *p <* 0.0001 (t-test). (C) * *p* = 0.0404 (t-test).

**Fig 3:**
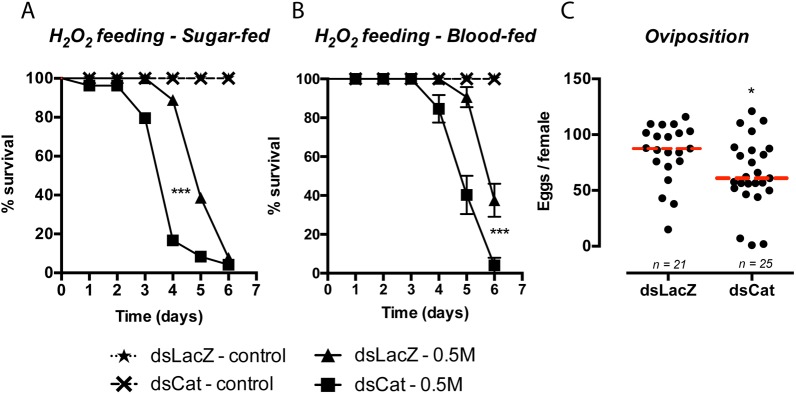
Catalase knockdown affected both resistance to H_2_O_2_ and oviposition. Catalase was silenced as described, and two days after dsRNA injection, SF (A) or BF (immediately after feeding) (B) mosquitoes were transferred to cages containing 5% sucrose supplemented with 1 M H_2_O_2_ *ad libitum* (day 0). A fresh H_2_O_2_ solution was provided daily. Survival was scored every 24 h. *** *p* < 0.0001 for the comparison between dsLacZ–H_2_O_2_ vs dsCat–H_2_O_2_ (log-rank test). (C) Catalase-silenced mosquitoes were blood-fed and allowed to lay eggs. Each dot represents an individual mosquito. LacZ–n = 21. Catalase–n = 25. * *p =* 0.37 (t-test).

We injected *Aedes aegypti* females with dsRNA against catalase and evaluated the mRNA levels in the midgut epithelia. Fig 2A shows that the catalase transcripts were reduced by 93% and 86% in SF and BF mosquitoes 24 h after feeding. Consistent with the reduced mRNA levels, we observed a decrease in catalase activity in the epithelia of blood-fed mosquitoes (Fig 2B). To determine whether catalase silencing affected midgut redox metabolism, we measured H_2_O_2_ (the substrate of catalase and a diffusible ROS) released by epithelial cells. Fig 2C shows that catalase knockdown increased hydrogen peroxide levels leaked to the supernatant. Together, Fig 2A–2C confirm that the RNAi approach negatively impacted catalase activity and redox metabolism of the midgut. To address its physiological significance, we demonstrated a reduction in lifespan of both sugar-fed (SF) and blood-fed (BF) mosquitoes challenged with sucrose supplemented with H_2_O_2_ (Fig 3A and 3B). The median time to death was anticipated in 1 day in the dsCatalase group, which represents 15–20% of the lifespan of mosquitoes feeding on hydrogen peroxide under the conditions tested. We also observed a small but statistically significant 22% reduction in oviposition (Fig 3C), similar to what was reported for *Anopheles gambiae* and *Lutzomyia longipalpis* [12,13].

### Catalase silencing reduces Dengue, but not Zika, midgut infection prevalence

**Fig 4:**
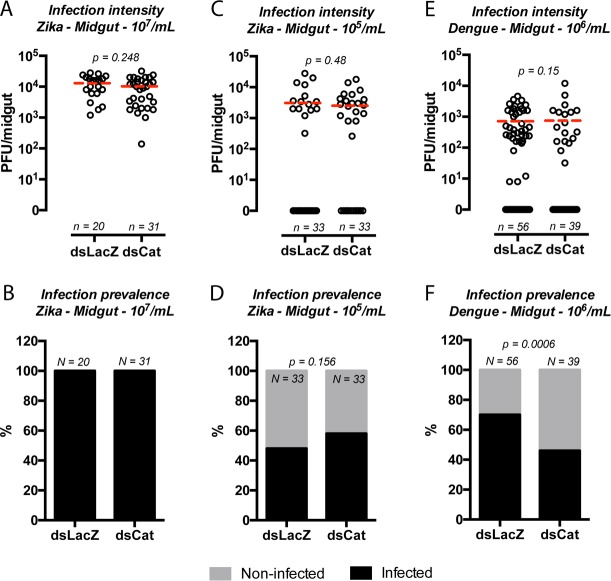
Catalase silencing impacted Dengue but not Zika midgut infection prevalence. (A) Females were fed blood contaminated with 10^7^ PFU/mL of Zika virus, and 7 days after feeding the number of PFU was determined in the midgut. (B) The percentage of infected midguts (infection prevalence) was scored from the same set of data as in A. (C) Mosquitoes were fed a lower dose of Zika-infected blood (10^5^/mL), and PFU/midgut was determined 7 days post-infection. (D) Infection prevalence of mosquitoes from C. (E) Mosquitoes were fed blood contaminated with 10^6^ PFU/mL of Dengue 4 virus, and PFU/midgut was counted 7 DPI. (F) Infection prevalence was determined from the same group of mosquitoes. Mann-Whitney *U*-tests were used for infection intensity (A, C, E), and chi-square tests were performed to determine the significance of infection prevalence analysis (B, D, F). Statistical values and number of replicates are depicted in the corresponding figures.

To test the hypothesis that antioxidant protection triggered by a blood meal could influence a mosquito’s infection status with different flavivirues, we challenged catalase-silenced *Aedes aegypti* with two doses of Zika virus and measured the number of PFU per midgut (infection intensity) and the number of infected midguts (infection prevalence) seven days after administration of virus-contaminated blood. Catalase silencing did not change any of the parameters evaluated in Zika-infected females. (Fig 4A–4D). Interestingly, the highest dose offered, 10^7^/mL (corresponding to our maximal titer obtained from C6/36 cells supernatants), and its 100-fold dilution, (10^5^/mL), resulted in only a four-fold change viral loads after 7 days (10^7^/mL mean PFU ~ 10000; 10^5^/mL mean PFU ~ 3000) (Fig 4A and 4C). However, the same doses resulted in a change in infection prevalence (10^7^/mL Zika viral particles produced 100% prevalence in both dsLacZ and dsCat while 10^5^/mL viral particles produced ~50% prevalence in both dsLacZ and dsCat) (Fig 4B and 4D). When we challenged dsCatalase-treated mosquitoes with the maximal Dengue-4 infectious dose (5 x 10^6^/mL), there was no alteration in the median infection intensity (Fig 4E). However, we observed a significant reduction in infection prevalence (70% of dsLacZ mosquitoes were infected vs 46% of dsCatalase; p = 0.0006 ±chisquare, demonstrating that a reduction in epithelial H_2_O_2_-removing capacity through catalase knockdown reduced the ability of Dengue-4, but not Zika virus, to infect the midgut of *Aedes aegypti*.

## Discussion

Overall, we showed that a blood meal induced antioxidant protection in the midgut of *Aedes aegypti* and that RNAi-mediated knockdown of catalase resulted in reduced oviposition and lifespan when mosquitoes were challenged with H_2_O_2_ and decreased midgut virus prevalence after infection with Dengue-4, but not Zika.

The levels of pro-oxidant molecules, including hydrogen peroxide, in a given tissue must be carefully monitored to maintain normal cellular functions, with deviations from the optimal concentration being potentially harmful for the organism. The increase in H_2_O_2_ levels may be particularly deleterious to hematophagous arthropods because blood digestion releases heme, a pro-oxidant molecule, which may interact with cellular ROS, leading to oxidative stress [30,38], decreased reproductive output [12,13] and possibly death of the insect [39,40]. We recently demonstrated that after a blood meal, *Aedes aegypti* inhibits the metabolic generation of ROS [24]. In that report, we showed that after blood intake, heme triggers a protein kinase C-dependent mechanism that inhibits dual oxidase (Duox) activity (a source of ROS in the midgut), maintaining low ROS production during blood digestion compared with SF levels. The pattern of antioxidant enzyme expression observed in most organisms shows that they typically respond to increased levels of ROS, such as catalase in *Rhodnius prolixus* [29]. It is peculiar, then, that *Aedes aegypti* increases catalase expression and activity after a blood meal, especially when ROS levels were reduced compared with sugar-fed mosquitoes. We hypothesize that hematophagous mosquitoes evolved a redundant protection strategy to prevent oxidative stress following blood intake, which may explain the simultaneous decrease in ROS production and increase in antioxidant capacity. This is a major departure from most studies on ROS metabolism, where antioxidant enzymes are regulated by previous oxidant stress.

Redox metabolism has been implicated in the response of *Anopheles* mosquitoes to plasmodium infection [20,41]. While in *Anopheles gambiae* catalase knockdown reduced *Plasmodium berghei* oocyst counts, supposedly through augmented concentration of toxic H_2_O_2_, it increased oocysts of the human malaria *P*. *vivax* in its natural vector, *Anopheles aquasalis* [22], revealing a complex and species-specific role of catalase in the gut of mosquitoes during malaria infection. Regarding the interaction of ROS and microorganisms, high levels of free radicals are believed to be detrimental. However, an emerging concept posits that similar to the role of catalase in *P*. *vivax–An*. *aquasalis*, other parasites, such as *Trypanosoma cruzi*, thrive under host oxidative stress [42–45]. In the case of *Aedes aegypti*, altering redox homeostasis in the gut through catalase silencing reduced Dengue prevalence in the midgut (Fig 4F), suggesting that ROS could antagonize infection by this specific arbovirus. However, catalase RNAi did not alter the percentage of infected midguts in females challenged with Zika (Fig 4B and 4D), indicating a differential sensitivity of flaviviruses to ROS produced by *Aedes aegypti*.

Our understanding of mosquito immunity to dengue virus has predominantly focused on classical immune genes and the roles of the Toll, Stat and RNAi pathways have been firmly established [46–49]. Additionally, genes that conventionally are not considered (or labeled) immune genes, such as lipid and redox metabolism, are also known to influence mosquito-arbovirus interactions and should not be overlooked [8,27,50]. Little is known about the molecular aspects of the interaction between *Aedes aegypti* and Zika, and antiviral mechanisms described for other flaviviruses will likely be involved [51]. In agreement with our result using Dengue-4, it was recently shown for Dengue-2 that RNAi-silencing of the ROS-producing enzymes Duox and NoxM, as well as treatment of mosquitoes with the antioxidant vitamin C, enhanced viral infection in *Aedes aegypti* [52]. The data presented here suggested that catalase silencing altered the so-called midgut infection barrier (MIB) for Dengue-4. MIB is a concept that refers to mechanisms involved in the inhibition of the initial contacts between virions and the intestinal epithelial cells, preventing the establishment of the infection [53,54]. These mechanisms may involve physical barriers, such as the peritrophic matrix, or physiological and immunological mechanisms, such as the midgut microbiota and/or the RNAi pathway, to name a few [55]. Here, catalase silencing was shown to reduce Dengue-4 prevalence possibly through an alteration in the midgut threshold of infection. If the virus is able to pass this bottleneck, then it establishes a successful cycle of replication, which was seen by the similar infection intensities of the catalase-silenced and control groups of insects. Importantly, this mechanism did not alter Zika prevalence in the midgut, indicating significant differences between how these two flaviviruses establish infections in the mosquito gut.

## References

[1] Gulland A. Zika virus is a global public health emergency, declares WHO. BMJ. 2016;352: i657 doi: 10.1136/bmj.i657 26839247 10.1136/bmj.i657

[2] Messina JP, Kraemer MUG, Brady OJ, Pigott DM, Shearer FM, Weiss DJ, et al Mapping global environmental suitability for Zika virus. Elife. 2016;5: 1–19. 10.7554/eLife.15272 PMC4889326 27090089

[3] Bhatt S, Gething PW, Brady OJ, Messina JP, Farlow AW, Moyes CL, et al The global distribution and burden of dengue. Nature. Nature Publishing Group; 2013;496: 504–507. doi: 10.1038/nature12060 23563266 10.1038/nature12060 PMC3651993

[4] Moreira LA, Iturbe-Ormaetxe I, Jeffery JA, Lu G, Pyke AT, Hedges LM, et al A Wolbachia Symbiont in Aedes aegypti Limits Infection with Dengue, Chikungunya, and Plasmodium. Cell. 2009;139: 1268–1278. doi: 10.1016/j.cell.2009.11.042 20064373 10.1016/j.cell.2009.11.042

[5] Dutra HLC, Rocha MN, Dias FBS, Mansur SB, Caragata EP, Moreira LA. Wolbachia Blocks Currently Circulating Zika Virus Isolates in Brazilian Aedes aegypti Mosquitoes. Cell Host Microbe. The Authors; 2016;19: 771–774. doi: 10.1016/j.chom.2016.04.021 27156023 10.1016/j.chom.2016.04.021 PMC4906366

[6] Harris AF, Nimmo D, McKemey AR, Kelly N, Scaife S, Donnelly CA, et al Field performance of engineered male mosquitoes. Nat Biotechnol. Nature Publishing Group; 2011;29: 1034–1037. doi: 10.1038/nbt.2019 22037376 10.1038/nbt.2019

[7] Winskill P, Carvalho DO, Capurro ML, Alphey L, Donnelly CA, McKemey AR. Dispersal of Engineered Male Aedes aegypti Mosquitoes. PLoS Negl Trop Dis. 2015;9: 1–18. 10.1371/journal.pntd.0004156 PMC4640874 26554922

[8] Sim S, Jupatanakul N, Ramirez JL, Kang S, Romero-Vivas CM, Mohammed H, et al Transcriptomic Profiling of Diverse Aedes aegypti Strains Reveals Increased Basal-level Immune Activation in Dengue Virus-refractory Populations and Identifies Novel Virus-vector Molecular Interactions. PLoS Negl Trop Dis. 2013;7. 10.1371/journal.pntd.0002295 PMC3701703 23861987

[9] Chouin-Carneiro T, Vega-Rua A, Vazeille M, Yebakima A, Girod R, Goindin D, et al Differential Susceptibilities of Aedes aegypti and Aedes albopictus from the Americas to Zika Virus. PLoS Negl Trop Dis. 2016;10: 1–11. 10.1371/journal.pntd.0004543 PMC4777396 26938868

[10] Janssen-Heininger YMW, Mossman BT, Heintz NH, Forman HJ, Kalyanaraman B, Finkel T, et al Redox-based regulation of signal transduction: Principles, pitfalls, and promises. Free Radic Biol Med. 2008;45: 1–17. doi: 10.1016/j.freeradbiomed.2008.03.011 18423411 10.1016/j.freeradbiomed.2008.03.011 PMC2453533

[11] Jones DP. Redefining Oxidative Stress. ANTIOXIDANTS REDOX Signal Vol 8, Numbers. 2006;8: 1–16. 10.1089/ars.2006.8.1865 16987039

[12] DeJong RJ, Miller LM, Molina-Cruz A, Gupta L, Kumar S, Barillas-Mury C. Reactive oxygen species detoxification by catalase is a major determinant of fecundity in the mosquito Anopheles gambiae. Proc Natl Acad Sci U S A. 2007;104: 2121–2126. doi: 10.1073/pnas.0608407104 17284604 10.1073/pnas.0608407104 PMC1892935

[13] Diaz-Albiter H, Mitford R, Genta FA, Sant’Anna MR V, Dillon RJ. Reactive oxygen species scavenging by catalase is important for female Lutzomyia longipalpis fecundity and mortality. PLoS One. 2011;6: 1–9. 10.1371/journal.pone.0017486 PMC3052318 21408075

[14] Ha E-M, Oh C-T, Bae YS, Lee W-J. A direct role for dual oxidase in Drosophila gut immunity. Science. 2005;310: 847–50. doi: 10.1126/science.1117311 16272120 10.1126/science.1117311

[15] Ha EM, Oh CT, Ryu JH, Bae YS, Kang SW, Jang I hwan, et al An antioxidant system required for host protection against gut infection in Drosophila. Dev Cell. 2005;8: 125–132. doi: 10.1016/j.devcel.2004.11.007 15621536 10.1016/j.devcel.2004.11.007

[16] Kumar S, Christophides GK, Cantera R, Charles B, Han YS, Meister S, et al The role of reactive oxygen species on Plasmodium melanotic encapsulation in Anopheles gambiae. Proc Natl Acad Sci U S A. 2003;100: 14139–14144. doi: 10.1073/pnas.2036262100 14623973 10.1073/pnas.2036262100 PMC283559

[17] Kumar S, Molina-Cruz A, Gupta L, Rodrigues J, Barillas-Mury C. A Peroxidase / Dual Oxidase System Modulates Epithelial Immunity in Anopheles gambiae. Science (80-). 2010;327: 1644–1649. doi: 10.1126/science.1184008 20223948 10.1126/science.1184008 PMC3510679

[18] Oliveira JHM, Gonçalves RLS, Oliveira GA, Oliveira PL, Oliveira MF, Barillas-Mury C. Energy metabolism affects susceptibility of Anopheles gambiae mosquitoes to Plasmodium infection. Insect Biochem Mol Biol. Elsevier Ltd; 2011;41: 349–355. doi: 10.1016/j.ibmb.2011.02.001 21320598 10.1016/j.ibmb.2011.02.001 PMC3078167

[19] Cirimotich CM, Dong Y, Clayton AM, Sandiford SL, Souza-Neto JA, Mulenga M, et al Natural Microbe-Mediated Refractoriness to Plasmodium Infection in Anopheles gambiae. Science (80-). 2011;332: 855–858. doi: 10.1126/science.1201618 21566196 10.1126/science.1201618 PMC4154605

[20] Gonçalves RLS, Oliveira JHM, Oliveira GA, Andersen JF, Oliveira MF, Oliveira PL, et al Mitochondrial reactive oxygen species modulate mosquito susceptibility to Plasmodium infection. PLoS One. 2012;7. 10.1371/journal.pone.0041083 PMC3399787 22815925

[21] Oliveira GA., Lieberman J, Barillas-Mury C. Epithelial Nitration by a Peroxidase/NOX5 System Mediates Mosquito Antiplasmodial Immunity. Science (80). 2012;335: 856–859. doi: 10.1126/science.1209678 22282475 10.1126/science.1209678 PMC3444286

[22] Bahia AC, Oliveira JHM, Kubota MS, Araújo HRC, Lima JBP, Ríos-Velásquez CM, et al The Role of Reactive Oxygen Species in Anopheles aquasalis Response to Plasmodium vivax Infection. PLoS One. 2013;8: 1–10. 10.1371/journal.pone.0057014 PMC3575503 23441231

[23] Graça-Souza A V., Maya-Monteiro C, Paiva-Silva GO, Braz GRC, Paes MC, Sorgine MHF, et al Adaptations against heme toxicity in blood-feeding arthropods. Insect Biochem Mol Biol. 2006;36: 322–335. doi: 10.1016/j.ibmb.2006.01.009 16551546 10.1016/j.ibmb.2006.01.009

[24] Oliveira JHM, Gonçalves RLS, Lara FA, Dias FA, Gandara ACP, Menna-Barreto RFS, et al Blood meal-derived heme decreases ROS levels in the midgut of Aedes aegypti and allows proliferation of intestinal microbiota. PLoS Pathog. 2011;7. 10.1371/journal.ppat.1001320 PMC3060171 21445237

[25] Lima VLA, Dias F, Nunes RD, Pereira LO, Santos TSR, Chiarini LB, et al The antioxidant role of xanthurenic acid in the Aedes aegypti midgut during digestion of a blood meal. PLoS One. 2012;7: 1–8. 10.1371/journal.pone.0038349 PMC3372515 22701629

[26] Walter-Nuno AB, Oliveira MP, Oliveira MF, Gonçalves RL, Ramos IB, Koerich LB, et al Silencing of maternal heme-binding protein causes embryonic mitochondrial dysfunction and impairs embryogenesis in the blood sucking insect rhodnius prolixus. J Biol Chem. 2013;288: 29323–29332. doi: 10.1074/jbc.M113.504985 23986441 10.1074/jbc.M113.504985 PMC3795234

[27] Bottino-Rojas V, Talyuli OAC, Jupatanakul N, Sim S, Dimopoulos G, Venancio TM, et al Heme signaling impacts global gene expression, immunity and dengue virus infectivity in Aedes aegypti. PLoS One. 2015;10: 1–19. 10.1371/journal.pone.0135985 PMC4537099 26275150

[28] Lara FA, Pohl PC, Gandara AC, Ferreira JDS, Nascimento-Silva MC, Bechara GH, et al ATP binding cassette transporter mediates both heme and pesticide detoxification in tick midgut cells. PLoS One. 2015;10: 1–20. 10.1371/journal.pone.0134779 PMC4530934 26258982

[29] Gandara ACP, Oliveira JHM, Nunes RD, Goncalves RLS, Dias FA, Hecht F, et al Amino acids trigger down-regulation of superoxide via TORC pathway in the midgut of Rhodnius prolixus. Biosci Rep. 2016;36: e00321–e00321. doi: 10.1042/BSR20160061 26945025 10.1042/BSR20160061 PMC4832317

[30] Paes MC, Oliveira MB, Oliveira PL. Hydrogen peroxide detoxification in the midgut of the blood-sucking insect, Rhodnius prolixus. Arch Insect Biochem Physiol. 2001;48: 63–71. doi: 10.1002/arch.1058 11568965 10.1002/arch.1058

[31] Balla J, Vercellotti GM, Jeney V, Yachie A, Varga Z, Jacob HS, et al Heme, heme oxygenase, and ferritin: how the vascular endothelium survives (and dies) in an iron-rich environment. Antioxid Redox Signal. 2007;9: 2119–2137. doi: 10.1089/ars.2007.1787 17767398 10.1089/ars.2007.1787

[32] Aebi H. Oxygen Radicals in Biological Systems. Methods Enzymol. 1984;105: 121–126. 6727658

[33] Lowry OH, Rosebrough NJ, Farr AL, Randall RJ. Protein measurement with the folin phenol reagent. J Biol Chem. 1951;193: 265–275. 14907713

[34] Gentile C, Lima JBP, Peixoto AA. Isolation of a fragment homologous to the rp49 constitutive gene of Drosophila in the Neotropical malaria vector Anopheles aquasalis (Diptera: Culicidae). Mem Inst Oswaldo Cruz. 2005;100: 545–547. doi: /S0074-02762005000600008 16302065 10.1590/s0074-02762005000600008

[35] Livak KJ, Schmittgen TD. Analysis of relative gene expression data using real-time quantitative PCR and. Methods. 2001;25: 402–408. doi: 10.1006/meth.2001.1262 11846609 10.1006/meth.2001.1262

[36] Noriega FG, Wells MA. A molecular view of trypsin synthesis in the midgut of Aedes aegypti. J Insect Physiol. 1999;45: 613–620. 12770346 10.1016/s0022-1910(99)00052-9

[37] Switala J.; Loewen P.C. Diversity of properties among catalases. Arch Biochem Biophys. 2002;401: 145–154. Available: http://www.sciencedirect.com/science/article/pii/S0003986102000498 doi: 10.1016/S0003-9861(02)00049-8 12054464 10.1016/S0003-9861(02)00049-8

[38] Citelli M, Lara FA, Vaz I da S, Oliveira PL. Oxidative stress impairs heme detoxification in the midgut of the cattle tick, Rhipicephalus (Boophilus) microplus. Mol Biochem Parasitol. 2007;151: 81–88. doi: 10.1016/j.molbiopara.2006.10.008 17123644 10.1016/j.molbiopara.2006.10.008

[39] Magalhaes T, Brackney DE, Beier JC, Foy BD. Silencing an Anopheles gambiae catalase and sulfhydryl oxidase increases mosquito mortality after a blood meal. Arch Insect Biochem Physiol. 2008;68: 134–143. doi: 10.1002/arch.20238 18454489 10.1002/arch.20238 PMC2673501

[40] Sim C, Denlinger DL. Catalase and superoxide dismutase-2 enhance survival and protect ovaries during overwintering diapause in the mosquito Culex pipiens. J Insect Physiol. Elsevier Ltd; 2011;57: 628–634. doi: 10.1016/j.jinsphys.2011.01.012 21277308 10.1016/j.jinsphys.2011.01.012 PMC3104096

[41] Jaramillo-Gutierrez G, Molina-Cruz A, Kumar S, Barillas-Mury C. The Anopheles gambiae oxidation resistance 1 (OXR1) gene regulates expression of enzymes that detoxify reactive oxygen species. PLoS One. 2010;5: 1–9. 10.1371/journal.pone.0011168 PMC2887368 20567517

[42] Paiva CN, Feijó DF, Dutra FF, Carneiro VC, Freitas GB, Alves LS, et al Oxidative stress fuels Trypanosoma cruzi infection in mice. J Clin Invest. 2012;122: 2531–42. doi: 10.1172/JCI58525 22728935 10.1172/JCI58525 PMC3386808

[43] Paiva CN, Bozza MT. Are reactive oxygen species always detrimental to pathogens? Antioxid Redox Signal. 2014;20: 1000–37. doi: 10.1089/ars.2013.5447 23992156 10.1089/ars.2013.5447 PMC3924804

[44] de Almeida Nogueira NP, de Souza CF, de Souza Saraiva FM, Sultano PE, Dalmau SR, Bruno RE, et al Heme-Induced ROS in Trypanosoma cruzi activates Camkii-Like that triggers epimastigote proliferation. One helpful effect of ROS. PLoS One. 2011;6. 10.1371/journal.pone.0025935 PMC3191175 22022475

[45] Nogueira NP, Saraiva FMS, Sultano PE, Cunha PRBB, Laranja GAT, Justo GA, et al Proliferation and differentiation of Trypanosoma cruzi inside its vector have a new trigger: Redox status. PLoS One. 2015;10: 1–16. 10.1371/journal.pone.0116712 PMC4324650 25671543

[46] Xi Z, Ramirez JL, Dimopoulos G. The Aedes aegypti toll pathway controls dengue virus infection. PLoS Pathog. 2008;4. 10.1371/journal.ppat.1000098 PMC2435278 18604274

[47] Souza-Neto JA, Sim S, Dimopoulos G. An evolutionary conserved function of the JAK-STAT pathway in anti-dengue defense. Proc Natl Acad Sci U S A. 2009;106: 17841–6. doi: 10.1073/pnas.0905006106 19805194 10.1073/pnas.0905006106 PMC2764916

[48] Sánchez-Vargas I, Scott JC, Poole-Smith BK, Franz AWE, Barbosa-Solomieu V, Wilusz J, et al Dengue virus type 2 infections of Aedes aegypti are modulated by the mosquito’s RNA interference pathway. PLoS Pathog. 2009;5. 10.1371/journal.ppat.1000299 PMC2633610 19214215

[49] Sim S, Jupatanakul N, Dimopoulos G. Mosquito immunity against arboviruses. Viruses. 2014;6: 4479–4504. doi: 10.3390/v6114479 25415198 10.3390/v6114479 PMC4246235

[50] Barletta ABF, Alves LR, Nascimento Silva MCL, Sim S, Dimopoulos G, Liechocki S, et al Emerging role of lipid droplets in Aedes aegypti immune response against bacteria and Dengue virus. Sci Rep. Nature Publishing Group; 2016;6: 19928 doi: 10.1038/srep19928 26887863 10.1038/srep19928 PMC4757862

[51] Colpitts TM, Cox J, Vanlandingham DL, Feitosa FM, Cheng G, Kurscheid S, et al Alterations in the aedes aegypti transcriptome during infection with west nile, dengue and yellow fever viruses. PLoS Pathog. 2011;7. 10.1371/journal.ppat.1002189 PMC3164632 21909258

[52] Liu J, Liu Y, Nie K, Du S, Qiu J, Pang X, et al Flavivirus NS1 protein in infected host sera enhances viral acquisition by mosquitoes. Nat Microbiol. Nature Publishing Group; 2016;1: 16087 doi: 10.1038/nmicrobiol.2016.87 27562253 10.1038/nmicrobiol.2016.87 PMC5003325

[53] Black WC IV, Bennett KE, Gorroch??tegui-Escalante N, Barillas-Mury C V., Fernandez-Salas I, Munoz MDL, et al Flavivirus susceptibility in Aedes aegypti. Arch Med Res. 2002;33: 379–388. 12234528 10.1016/s0188-4409(02)00373-9

[54] Franz AWE, Kantor AM, Passarelli AL, Clem RJ. Tissue barriers to arbovirus infection in mosquitoes. Viruses. 2015;7: 3741–3767. doi: 10.3390/v7072795 26184281 10.3390/v7072795 PMC4517124

[55] Kramer LD, Ciota AT. Dissecting vectorial capacity for mosquito-borne viruses. Curr Opin Virol. Elsevier B.V.; 2015;15: 112–118. doi: 10.1016/j.coviro.2015.10.003 26569343 10.1016/j.coviro.2015.10.003 PMC4688158

